# RNA-Seq analysis reveals the important co-expressed genes associated with polyphyllin biosynthesis during the developmental stages of *Paris polyphylla*

**DOI:** 10.1186/s12864-022-08792-2

**Published:** 2022-08-05

**Authors:** Xiaoyang Gao, Qixuan Su, Jing Li, Wenjing Yang, Baolin Yao, Jiawei Guo, Shengying Li, Changning Liu

**Affiliations:** 1 CAS Key Laboratory of Tropical Plant Resources and Sustainable Use, Xishuangbanna Tropical Botanical Garden, Chinese Academy of Sciences, Kunming, 650223 Yunnan China; 2 School of Life Sciences, University of Science and Technology of China, Hefei, 230026 Anhui China; 3 Spice and Beverage Research Institute, Chinese Academy of Tropical Agricultural Sciences, Wanning, 571533 Hainan China; 4 College of Life Sciences, University of Chinese Academy of Sciences, Beijing, 100049 China; 5 State Key Laboratory of Microbial Technology, Shandong University, Qingdao, 266237 Shandong China; 6 Center of Economic Botany, Core Botanical Gardens, Chinese Academy of Sciences, Menglun, 666303 Mengla, Yunnan China; 7 The Innovative Academy of Seed Design, Chinese Academy of Sciences, Kunming, 650223 Yunnan China

**Keywords:** Polyphyllin concentration, Spatiotemporal gene expression, Developmental stage, Polyphyllin biosynthesis, Gene co-expression network

## Abstract

**Background:**

Plants synthesize metabolites to adapt to a continuously changing environment. Metabolite biosynthesis often occurs in response to the tissue-specific combinatorial developmental cues that are transcriptionally regulated. Polyphyllins are the major bioactive components in *Paris* species that demonstrate hemostatic, anti-inflammatory and antitumor effects and have considerable market demands. However, the mechanisms underlying polyphyllin biosynthesis and regulation during plant development have not been fully elucidated.

**Results:**

Tissue samples of *P. polyphylla* var. *yunnanensis* during the four dominant developmental stages were collected and investigated using high-performance liquid chromatography and RNA sequencing. Polyphyllin concentrations in the different tissues were found to be highly dynamic across developmental stages. Specifically, decreasing trends in polyphyllin concentration were observed in the aerial vegetative tissues, whereas an increasing trend was observed in the rhizomes. Consistent with the aforementioned polyphyllin concentration trends, different patterns of spatiotemporal gene expression in the vegetative tissues were found to be closely related with polyphyllin biosynthesis. Additionally, molecular dissection of the pathway components revealed 137 candidate genes involved in the upstream pathway of polyphyllin backbone biosynthesis. Furthermore, gene co-expression network analysis revealed 74 transcription factor genes and one transporter gene associated with polyphyllin biosynthesis and allocation.

**Conclusions:**

Our findings outline the framework for understanding the biosynthesis and accumulation of polyphyllins during plant development and contribute to future research in elucidating the molecular mechanism underlying polyphyllin regulation and accumulation in *P. polyphylla*.

**Supplementary Information:**

The online version contains supplementary material available at 10.1186/s12864-022-08792-2.

## Background

*Paris polyphylla* Sm. is a perennial medicinal herb mainly distributed in Southwest China and sporadically in Burma, and other pan-Himalayan regions [1, 2]. The dried rhizomes of *P. polyphylla* varieties, generally called Rhizoma Paridis were first recorded as “Zaoxiu” in the “Divine Farmer’s Classic of Materia Medica” and they have been widely used in traditional Chinese medicine (TCM) for more than 2,000 years [1]. According to modern pharmacological studies, *P. polyphylla* has multiple medicinal properties, including hemostatic, anti-inflammatory, antimicrobial, and antitumor effects [3]. Currently, Rhizoma Paridis is the principal ingredient of over 80 kinds of patented medicines, boosting its local and international trade [4]. In addition, approximately 210 chemical components, including steroidal saponins, flavonoids, cholestanols, and molting hormones, have been verified in *P. polyphylla* and several other *Paris* species [5, 6]. To date, more than 120 steroidal saponins, i.e. polyphyllins have been identified, accounting for approximately 57% of the total number of known bioactive compounds in *Paris* species. Polyphyllin I, II, VI, and VII are considered as the key polyphyllins that have been established as the authoritative quality standards for the pharmacological components in *P. polyphylla* [7]. Notably, a 400-fold increase in the market price of *P. polyphylla* rhizomes has been observed in the past 40 years, of which 800–1050 metric tons of *P. polyphylla* rhizomes was sold annually [4]. However, the resources of wild *P. polyphylla* are being threatened by plundering exploitation caused by the increasing demand for herbal medicines with substantial economic value [8]. As *P. polyphylla* is a non-model plant, studies focused on investigating the polyphyllin biosynthetic genes (PBGs) and transcription regulators are limited. Therefore, molecular studies on this vulnerable medicinal herb are necessary to alleviate the resource shortage and to assist in creating methods for its future sustainability.

In the recent years, *P. polyphylla* varieties have been attracted a growing attention because of their medicinal and economic significance. However, their secondary seed dormancy, slow growth rate, and overexploitation of natural resources have directly resulted in plant resource shortage. To overcome these agricultural production problems, previous studies have focused on seed dormancy and germination [9], growth and development [10], artificial cultivation [11], quality evaluation [12], and genetic diversity and conservation [13]. A considerable progress has been made in the phytochemical analysis, particularly in determining the relative activities of newly identified polyphyllins and other bioactive compounds [14, 15]. The exploration of PBGs has remarkably advanced via next-generation sequencing. For example, the upstream candidate genes of steroidal saponin biosynthesis have been predicted using the transcriptome data of rhizomes [16] or leaves (stems) [17] or a mixture of tissues [18]. Specifically, the gene encoding cytochrome P450 monooxygenase (P450), which catalyzes the oxidative 5,6-spiroketalization of cholesterol to produce diosgenin, has been identified [19]. Another P450 gene and a cycloartenol synthase (CAS) gene possibly associated with polyphyllin biosynthesis were determined through heterologous expression in yeast [20, 21]. However, irrespective of whether the biosynthetic genes identified in vitro or not, the relationships between the PBGs and plant development remain unclear. Several reports correlate polyphyllin concentration with the cultivation years [22, 23], and the distinct accumulation patterns in leaves and rhizomes during two developmental stages have been proposed [24]. Deciphering the relationships between polyphyllin production and plant development will improve the understanding of mechanisms underlying the biosynthesis of bioactive compounds. In plants, biosynthesis of specialized metabolites often occurs in response to tissue-specific combinatorial developmental cues that are transcriptionally regulated [25]. Currently, the molecular mechanisms regulating polyphyllin biosynthetic pathway and accumulation are relatively unknown, and limited genetic resources are available for *P. polyphylla*.

In the present study, we obtained transcriptomic and phytochemical data from both the vegetative and the reproductive tissues during four dominant developmental stages of *P. polyphylla* var. *yunnanensis*. Quantitative analysis of compounds revealed that polyphyllins are widely present in the studied tissues, with dynamic trends in concentration during developmental stages. Different accumulation patterns of polyphyllin in the vegetative tissues were also detected and then confirmed using subsequent RNA-Seq analysis. Furthermore, the regulatory network between PBGs and transcription factor (TF) genes was explored for the first time via gene co-expression network analysis (GCNA). The results indicated that polyphyllin biosynthesis is coordinately regulated by both PBGs and TFs. Our study provides additional *P. polyphylla* genetic data, and contributes to future research on molecular mechanisms underlying polyphyllin biosynthesis and regulation.

## Results

### Changes in polyphyllin concentration in tissues during plant development

**Fig. 1:**
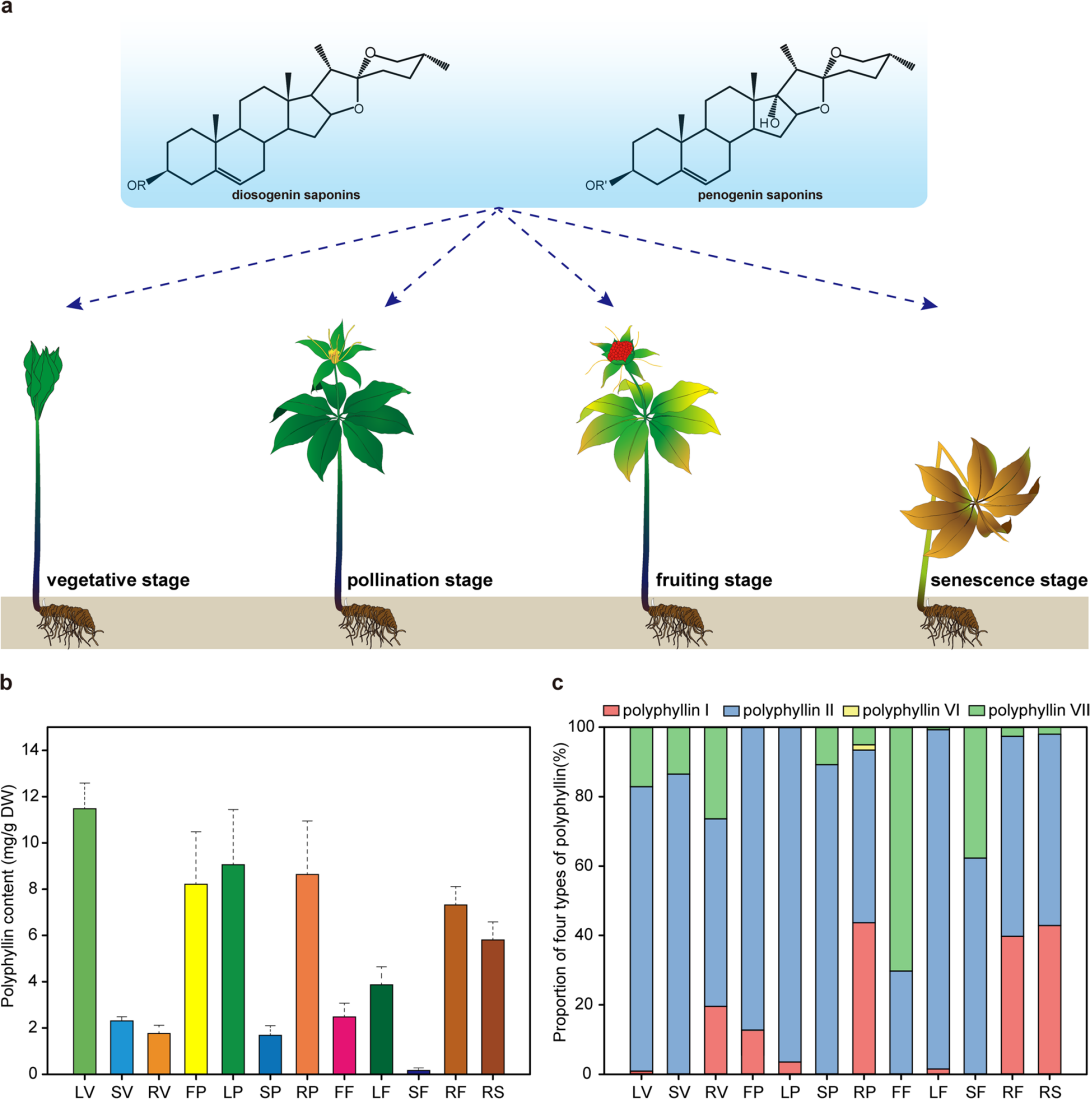
Schematic representation of this study design and polyphyllin concentration in different tissues. (**a**) Schematic diagram of *P. polyphylla* at four dominant developmental stages, including the vegetative stage, pollination stage, fruiting stage and senescence stage. (**b**) The changes in polyphyllin concentrations of twelve different tissues during the developmental stages. LV: leaves at the vegetative stage, SV: stems at the vegetative stage, RV: rhizomes at the vegetative stage; FP: flowers at the pollination stage, LP: leaves at the pollination stage, SP: stems at the pollination stage, RP: rhizomes at the pollination stage; FF: fruits at the fruiting stage, LF: leaves at the fruiting stage, SF: stems at the fruiting stage, RF: rhizomes at the fruiting stage; RS: rhizomes at the senescence stage. (**c**) The proportion of four types of polyphyllins in different tissues during development

Polyphyllin concentrations are highly dynamic during plant development, and they appear to be influenced by the ontogenetic trajectories of specific tissues. To determine polyphyllin concentration in *P. polyphylla* during development, the extracts from different tissues across four developmental stages were subjected to high-performance liquid chromatography (HPLC) (Fig. 1a). The highest polyphyllin concentration (11.48 mg/g DW) was detected in the leaves at the vegetative stage (Fig. 1b). The polyphyllin concentration in the leaves gradually decreased with development, and it was 3.87 mg/g DW at the fruiting stage. In the contrast, polyphyllin concentration in the stems was obviously lower than that in the leaves, but the decreasing trend in polyphyllin concentration was similar between the two organs. Notably, the variation of polyphyllin concentration in the rhizome was complex. Compared with that in its aerial counterparts, polyphyllin concentration in the rhizome was low at the vegetative stage, it peaked at the pollination stage, and remained relatively higher at the fruiting and senescence stages. Furthermore, polyphyllin concentration was higher in reproductive tissues (flowers and fruits) than vegetative tissues. The proportion of four key polyphyllins in the tissues also changed during plant development (Fig. 1c). Specifically, polyphyllin II accounted for the largest proportion in almost all the tissues. Polyphyllin II and VII were the major components in both the leaves and stems at vegetative stage. However, polyphyllin II concentration increased with leaf development, whereas polyphyllin VII concentration increased with stem development. Interestingly, more types of polyphyllins–Polyphyllin I, II and VII were mostly found in the rhizomes; however, only polyphyllin I concentration increased with rhizome development. These findings suggest that polyphyllin biosynthesis and accumulation occur in both the reproductive and vegetative tissues of *P. polyphylla*. Moreover, polyphyllin accumulation seemed to exhibit a tissue-specific regulation pattern depending on developmental stages.

### Transcriptome sequencing and differential expression analysis

To gain a comprehensive overview of the polyphyllin biosynthesis, twelve cDNA libraries prepared from the leaf, stem, rhizome, flower, and fruit tissues (with three biological replicates each) were sequenced using the Illumina HiSeq 2500 platform. Combined with RNA-Seq data of the leaves and rhizomes at the vegetative and the fruiting stages from our previous study, a total of 256.64 Gb clean data were obtained, with > 6 Gb data generated for each tissue sample (Additional file 1 Table S1). A set of 341,191 unigenes (N50 length: 688 bp and mean length: 564 bp) was generated by assembling high-quality reads and removing redundant transcript sequences, and the GC content was estimated 41.30%. In addition, the completeness of assembled transcriptome was analyzed with program BUSCO. On the basis of results, a total of 1440 single copy gene (total BUSCO groups searched), 72.4% of them perfectly mapped the complete transcripts, 12.6% of them fragmentally mapped the transcripts, 15.0% of them didn’t mapped any transcripts (Additional file 2 Figure S1). In short, good completeness and accuracy of assembled results revealed that RNA-Seq data in this study was sound and effective. Among all unigenes, 99,286 (29.10%), 57,572 (16.87%), 16,500 (4.87%), 65,359 (19.16%), 25,880 (7.59%), and 24,397 (7.15%) unigenes showed significant similarity to known proteins in NR, GO, KEGG, KOG, SwissProt, and Pfam databases, respectively (Additional file 1 Table S2). The assembled transcriptome together with annotation is provided in the supplementary files (Additional file 3). In addition, 29.76% of the unigenes were successfully annotated in at least one database, approximately 70.24% of them did not match with the entries in any of the six public databases. Majority (94.15%) of the unannotated unigenes were classified as noncoding RNA candidates by noncoding RNA prediction. The genome size of *P. polyphylla* was estimated to be > 50 Gb, but no high-quality genome sequences were previously reported under the order Liliales [26, 27]. Thus, more attention and efforts are required to annotate the assembled unigenes with unknown functions to contribute to the available genetic resource of *P. polyphylla*.

**Fig. 2:**
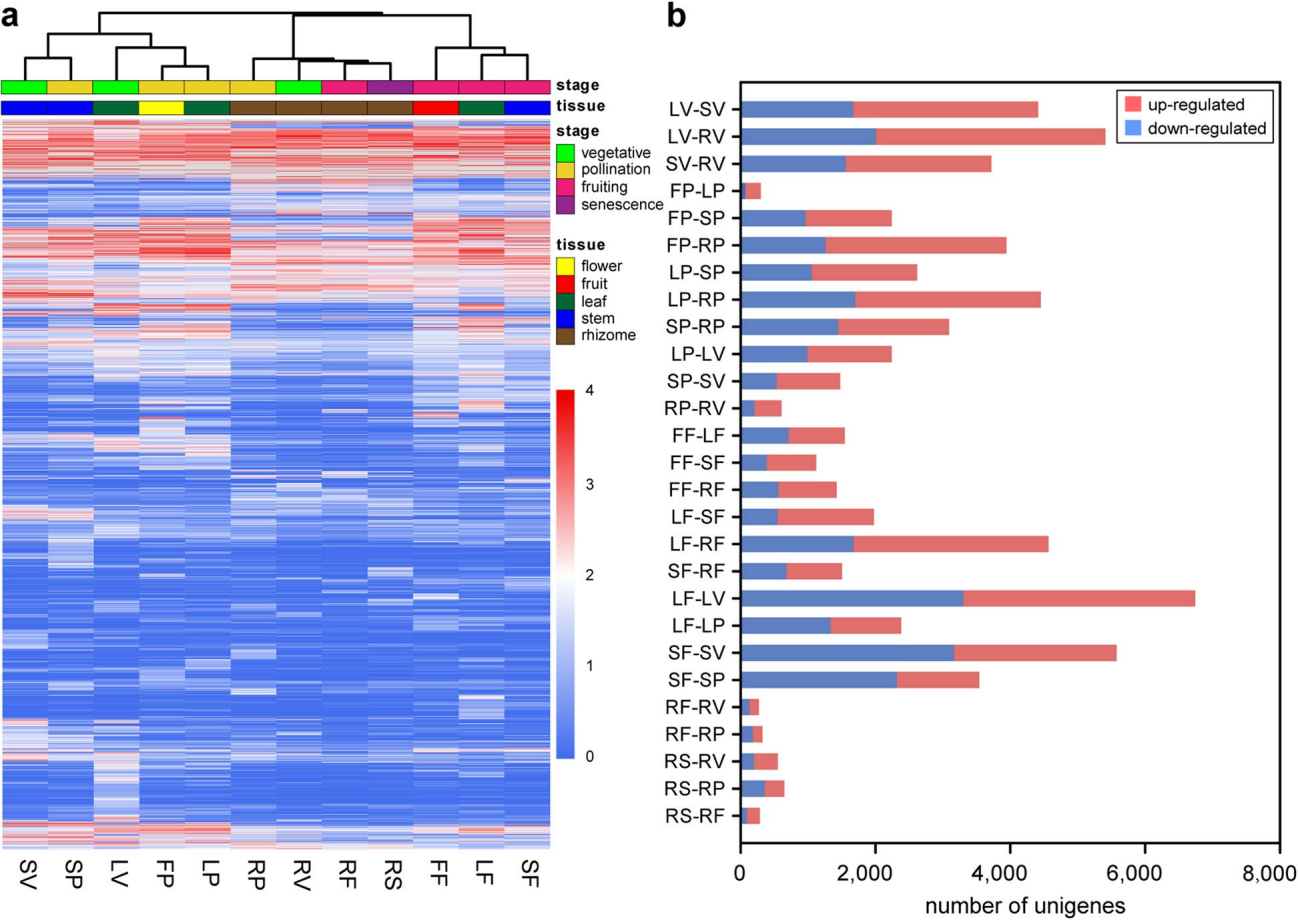
An overview of DEGs. (**a**) Gene expression profiles of 23,759 DEGs in 27 valid comparative paired-groups. The colored block under the branches represents the specific stage or tissue. The scale bar on the right represents gene expression levels. Blue and red denotes the low-level expression and high-level expression of the related genes. (**b**) The number of upregulated and downregulated DEGs. The red bar represents the up-regulated gene, and blue bar represents the down-regulated gene

The comparative transcriptomic analysis was performed to identify differentially expressed genes (DEGs) between the tissue samples. The comparisons were performed for the different tissues at a specific developmental stage and for a specific tissue at different developmental stages. In total, 23,579 DEGs were identified from 27 paired-groups. Hierarchical clustering of DEG expression profiles revealed that different tissues at the same developmental stage and a specific tissue across developmental stages exhibit similar gene expression patterns (Fig. 2a). Specifically, the DEGs in the aerial parts (leaf and stem) at the vegetative and the pollination stages clustered together, whereas those of the aerial parts at the fruiting stage clustered together. The DEGs in rhizomes across four development stages clustered together. Notably, the tissues in physically higher locations on the plant contained more upregulated genes than the tissues in lower positions (Fig. 2b). For instance, at the vegetative stage, more upregulated genes were identified in leaves than in stem, whereas more upregulated genes were identified in stems than in rhizomes. In addition, the number of upregulated genes in a specific tissue was different between developmental stages. Compared to vegetative and pollination stages, fewer upregulated DEGs were identified in the leaves and stems at the fruiting stage. By contrasting the rhizome at pollination stage, less upregulated genes were identified in the rhizomes during the other three stages (Fig. 2b).

**Fig. 3:**
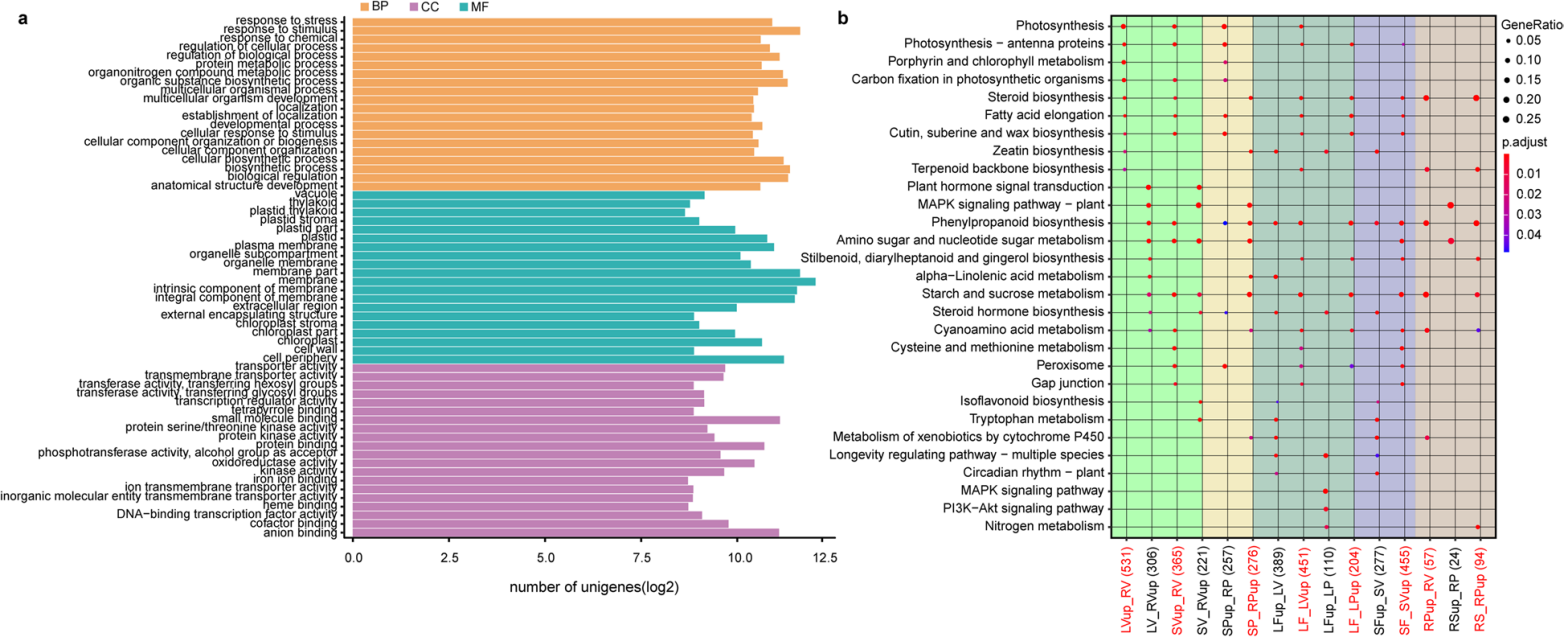
Functional enrichments of DEGs. (**a**) GO enrichment analysis of DEGs. (**b**) KEGG enrichment analysis of DEGs. The same tissue at different developmental stages is highlighted. The same stage containing different tissues is also highlighted. The scale bar on the right represents corrected *p*-value

To uncover the underlying differences in gene function, these DEGs were further subjected to GO and KEGG enrichment analysis. The top 20 significantly enriched GO terms in the three annotation categories are shown in Fig. 3a. The majority of DEGs were found to be involved in primary metabolic processes. Furthermore, some DEGs were evidently enriched in the biosynthetic pathways of secondary metabolites, thus revealing the biological functions of DEGs and complex biological behaviors of the transcriptome (Fig. 3b; Additional file 2 Figure S2). Genes associated with “isoflavonoid biosynthesis” and “trpytophan metabolism”, which are associated with environment stresses and plant hormone precursors, were highly enriched in the rhizomes during the vegetative stage and in the aerial tissues during the fruiting stage. Genes related to “photosynthesis”, “antenna protein”, and “carbon fixation” were undoubtedly enriched in the aerial vegetative tissues before the fruiting stage. Other functional genes were also highly enriched in specific tissues at specific developmental stages. For instance, genes related to “cysteine and methionine metabolism” and “gap junction” were only enriched in the leaves and the stems at the vegetative stage, whereas genes related to “longevity regulating pathway” were specifically enriched in leaves and stems at the fruiting stage. Genes from “fatty acid elongation” and “cutin, suberine and wax biosynthesis” were also enriched in the young leaves and stems before the fruiting stage. While the genes from “MAPK signaling pathway” were enriched in the rhizomes during the vegetative and pollination stages. Notably, the genes involved in steroid biosynthesis were significantly enriched in some specific tissues, including young aerial tissues (leaves at the vegetative and pollination stage, stems at the vegetative stage) and rhizomes at the pollination stage. Genes involved in terpenoid backbone biosynthesis, which is an important constituent part of steroid biosynthesis, were significantly enriched in the young leaves and rhizomes at the pollination stage. Thus, these findings suggest that the young aerial vegetative tissues before the fruiting stage and rhizomes at the pollination stage play important roles in polyphyllin biosynthesis.

### Determination of gene expression patterns and polyphyllin biosynthesis pathway

**Fig. 4:**
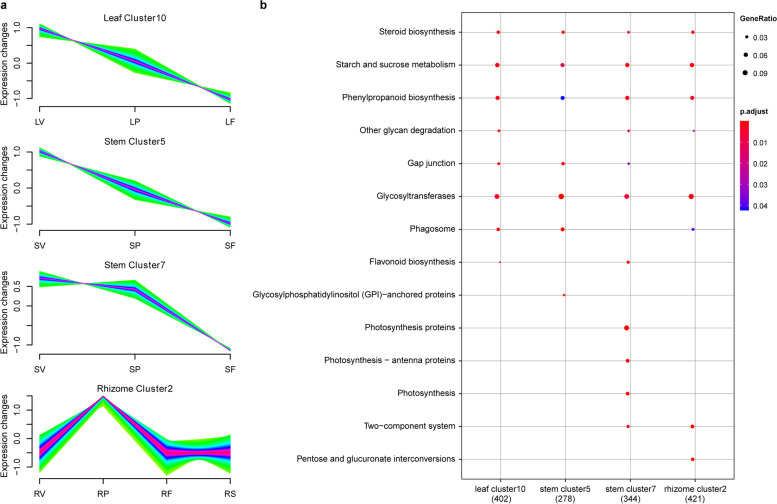
The gene expression patterns of tissues. (**a**) The gene expression patterns of leaf, stem, and rhizome. (**b**) The functional enrichment analysis of the four expression patterns of gene clusters. The scale bar on the right represents corrected *p*-value

To elucidate polyphyllin biosynthesis in tissues, the spatiotemporal gene expression patterns of the studied tissues were dissected. Ten main clusters were detected from the gene expression profiles of leaves, stems, and rhizomes across three or four stages (Additional file 2 Figures S3, S4 and S5). The expression patterns of four clusters, namely, cluster 10 from the leaves, cluster 5 and 7 from the stems, and cluster 2 from the rhizomes, were consistent with the changes in polyphyllin concentrations in the corresponding tissues (Fig. 4a). Cluster 10 from the leaves showed a gradually decrease in gene expression level with the development; this pattern was similarly observed in the stems. In contrast, the gene expression level of cluster 2 from the rhizomes augmented and peaked at the pollination stage. These results indicated that polyphyllin biosynthesis was activated in response to transcriptionally regulated combinatorial developmental cues. Notably, genes in the four clusters were primarily associated with polyphyllin biosynthesis (Fig. 4b). According to KEGG enrichment analysis, steroid biosynthesis-related genes were significantly enriched in these four clusters, suggesting that PBGs exhibit tissue-specific gene expression pattern depending on the developmental stages.

**Fig. 5:**
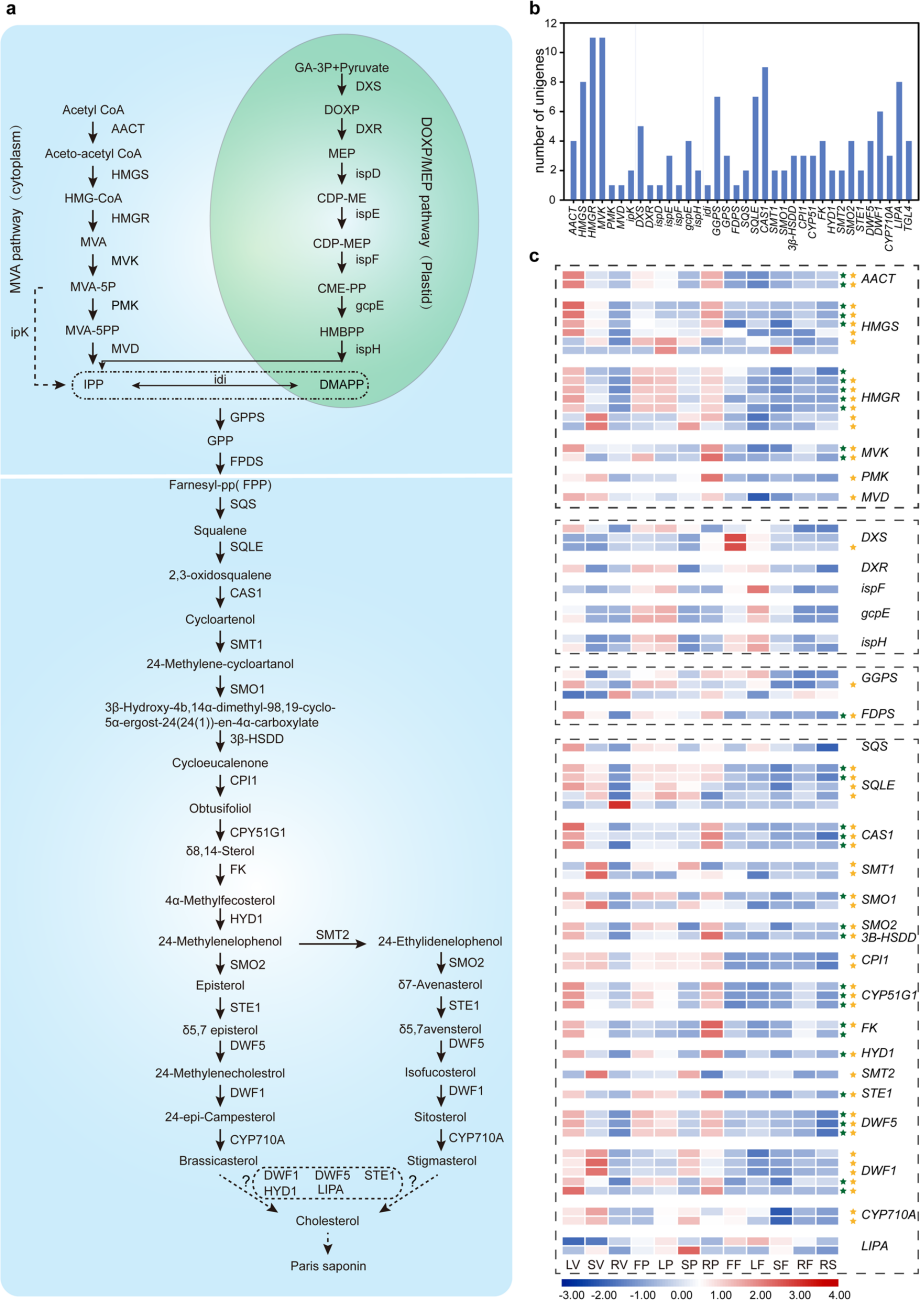
The biosynthetic pathway of polyphyllin and the identified PBGs. (**a**) The putative biosynthetic pathway. (**b**) The number of PBGs. (**c**) The expression profile of differentially expressed PBGs. The green star represents the PBGs identified in the target modules of co-expression network analysis. The yellow start represents the PBGs determined in the above spatiotemporal expression pattern. The scale bar on the bottom represents gene expression levels. Blue and red denotes the low-level expression and high-level expression of the related genes

In addition, several biosynthetic candidate genes were identified to delineate the polyphyllin biosynthesis pathway. A total of 137 PBG candidates potentially participated in the upstream pathway of polyphyllin biosynthesis (Fig. 5a and b). The majority of candidate genes related to the cytosolic mevalonate (MVA) pathway and the plastidial 2-C-methyl-D-erythritol 4-phosphate (MEP) pathway were also determined, which lead to squalene biosynthesis. Additionally, 69 PBGs were differentially expressed; half of these biosynthetic DEGs were found to be included in the cyan module and the darkturquoise module through GCNA using WGCNA program (Fig. 5c; Additional file 2 Figure S6a). The two modules, which were closely related to polyphyllin II and total of four polyphyllins, were discovered the involvement of DEGs in steroid biosynthesis (Additional file 2 Figure S6b). In addition, 52 biosynthetic DEGs were detected in the four spatiotemporal gene expression patterns mentioned above (Fig. 5c). The upstream genes for polyphyllin backbone biosynthesis, which were not supported by either modules or expression patterns, were mainly related to MEP pathway. In other words, the above PBGs supported by two analyses potentially encode the key enzymes involved in polyphyllin biosynthesis. However, the other candidate genes identified may also assist in polyphyllin biosynthesis, and therefore, they required further investigation. Taken together, our findings suggest that the expression patterns of PBGs are potentially in a tissue-specific and developmental stage-dependent way. The upstream pathway of polyphyllin biosynthesis was outlined, and the candidate genes for the further study were determined. We hypothesize that MEP pathway is possibly an auxiliary pathway for polyphyllin biosynthesis.

### Identification of TF and transporter genes co-expressed with PBGs

**Fig. 6:**
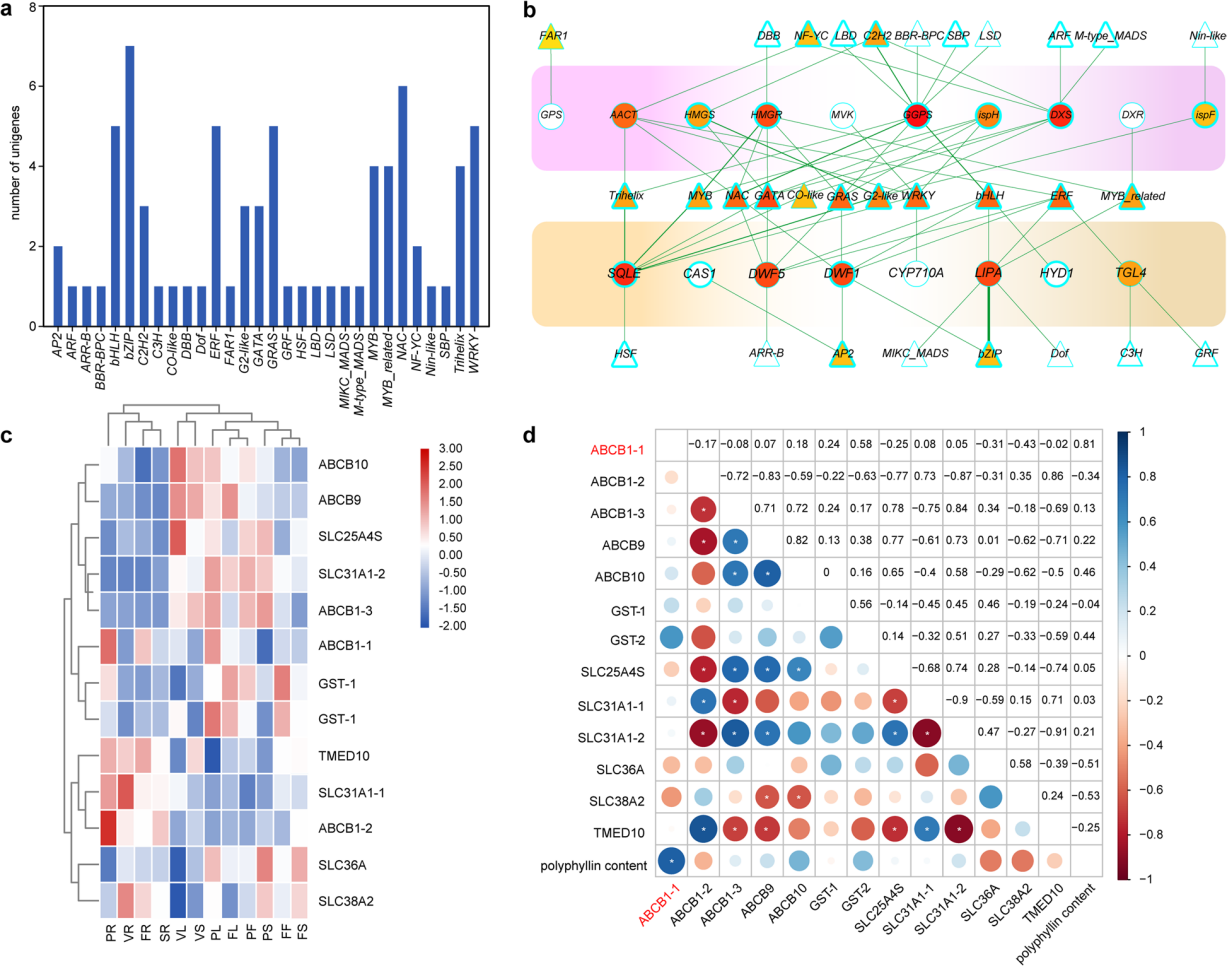
The regulated genes candidates involves in the polyphyllin biosynthesis. (**a**) The number of putative TF genes participated in the biosynthesis. (**b**) The relationships between TF genes and PBGs in the co-expression network. The circle denotes the PBG, and the triangle denotes the TF gene. The circle or triangle appeared in bold represent the DEG. The line thickness between PBG and TF gene represent occurrence frequency of co-expression. (**c**) The expression level of transporter genes co-expressed with the biosynthetic genes. The scale bar on the right represents gene expression levels. (**d**) The correlation between the above-mentioned transporter genes and polyphyllin concentration. The star represents the significance (*p* value < 0.05). The scale bar on the right represents the correlation between the expression level of transporter genes and polyphyllin content

To elucidate the transcriptional regulation of polyphyllin biosynthesis, TF genes co-expressed with PBGs were predicted by GCNA. A total of 74 TF genes probably to involved in polyphyllin biosynthesis were predicted (Fig. 6a). Moreover, transcriptional regulators and their putative target PBGs were illustrated in the gene co-expression network (Fig. 6b). Some TF genes that reportedly regulate the terpenoids biosynthesis, such as *NAC*, *ERF*, and *WRKY*, were highly co-expressed with PBGs. Given that the transporters can affect polyphyllin accumulation, the related transporter candidates were also predicted using GCNA. Compared with TF genes, only 13 of the 1,262 transporter candidate genes were identified to be co-expressed with PBGs. Specifically, 5 genes from the transporter system superfamily ATP-binding cassette (ABC), 5 genes from the solute carrier (SLC) transporter family, 2 genes from glutathione S-transferase (GST) family, and one gene from the transmembrane Emp24 domain-containing protein (TMED) family—co-expressed with PBGs. Expression levels of transporter genes in the different tissues were markedly varied when plotted using a heat map (Fig. 6c). The correlation analysis revealed a significant positive correlation (*p* value < 0.01) between the expression level of *ABCB1-1* and polyphyllin concentration (Fig. 6d). Hence, we speculate that *ABCB1-1* is a key transporter candidate gene in polyphyllin accumulation.

**Fig. 7:**
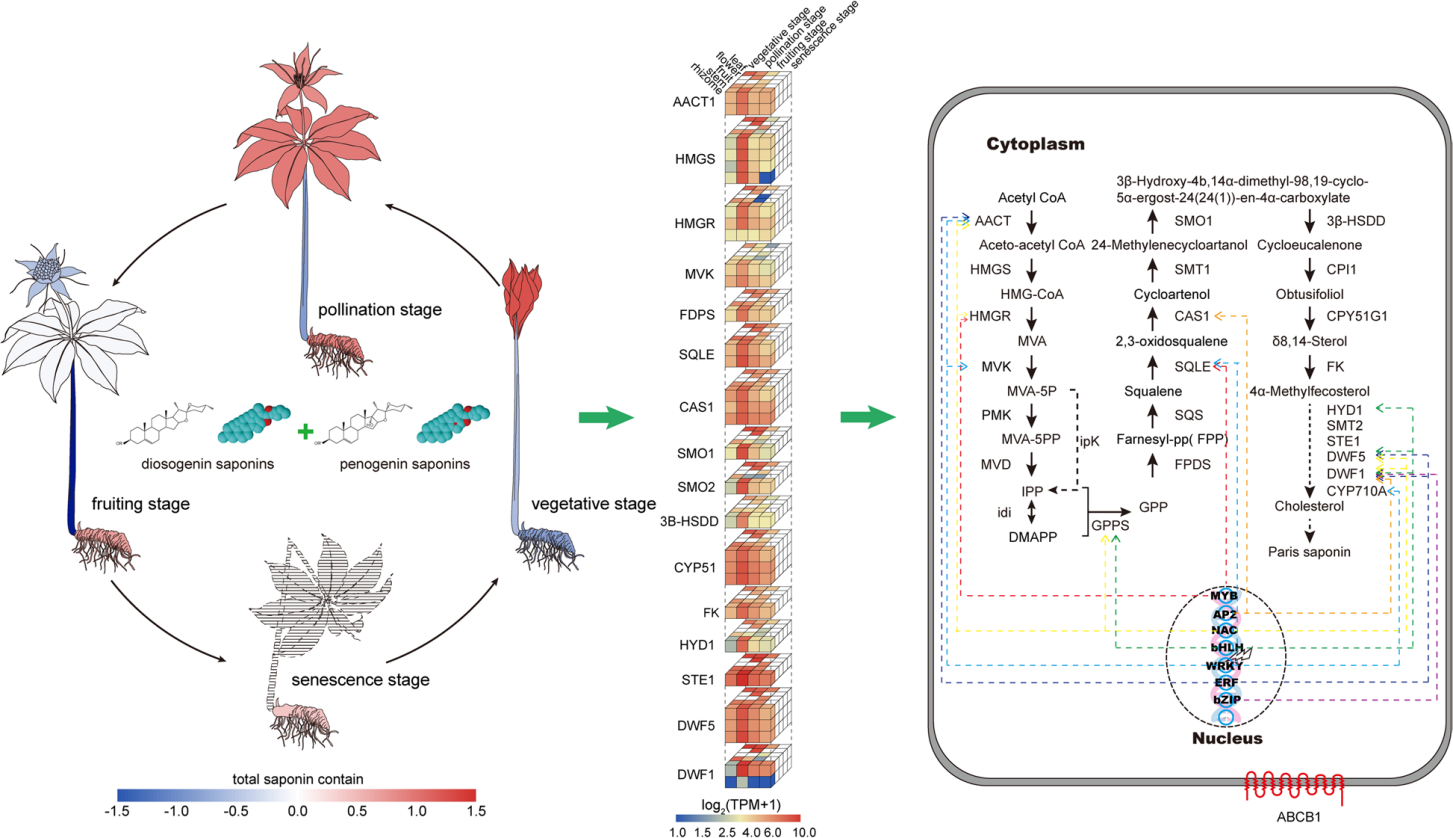
The schematic diagram of polyphyllin biosynthesis. The left diagram indicates the variations in polyphyllin content in *P. polyphylla*. The scale bar on the bottom of circle represents the polyphyllin content. The cubic heatmap in the middle indicates well-supported PBGs in the polyphyllin-related co-expression module and in spatiotemporal expression clusters. The scale bar on the bottom of the cube represents the expression level of biosynthetic genes. The right diagram indicates the transcriptional factors and transporters which may be associated polyphyllin biosynthesis and accumulation

A schematic diagram was drawn to understand polyphyllin biosynthesis and the underlying regulatory mechanism (Fig. 7). Polyphyllin concentration in different tissues of *P. polyphylla* var. *yunnanensis* constantly varied with plant development. Polyphyllin concentration in the aerial parts, specifically the leaves, gradually declined, while that in the rhizomes elevated and remained high even at the senescence stage. The cubic heat map showed that 31 unigenes encoded for 16 key enzymes in the upstream of polyphyllin biosynthesis pathway, which were observed in both the spatiotemporal gene expression patterns and the modules associated with polyphyllin. In particular, the PBGs in the rhizomes were highly expressed at the pollination stage, while those in the leaves were highly expressed at the vegetative stage and gradually decreased with development. Based on gene co-expression network of the TFs and PBGs, six typical TF gene types previously reported to be involved in triterpene saponin biosynthesis exhibited putative regulation. Additionally, the key candidate transporter for polyphyllin accumulation, ABCB1, was also observed in the schematic diagram.

### Quantitative reverse transcription PCR (qRT-PCR) validation

**Fig. 8:**
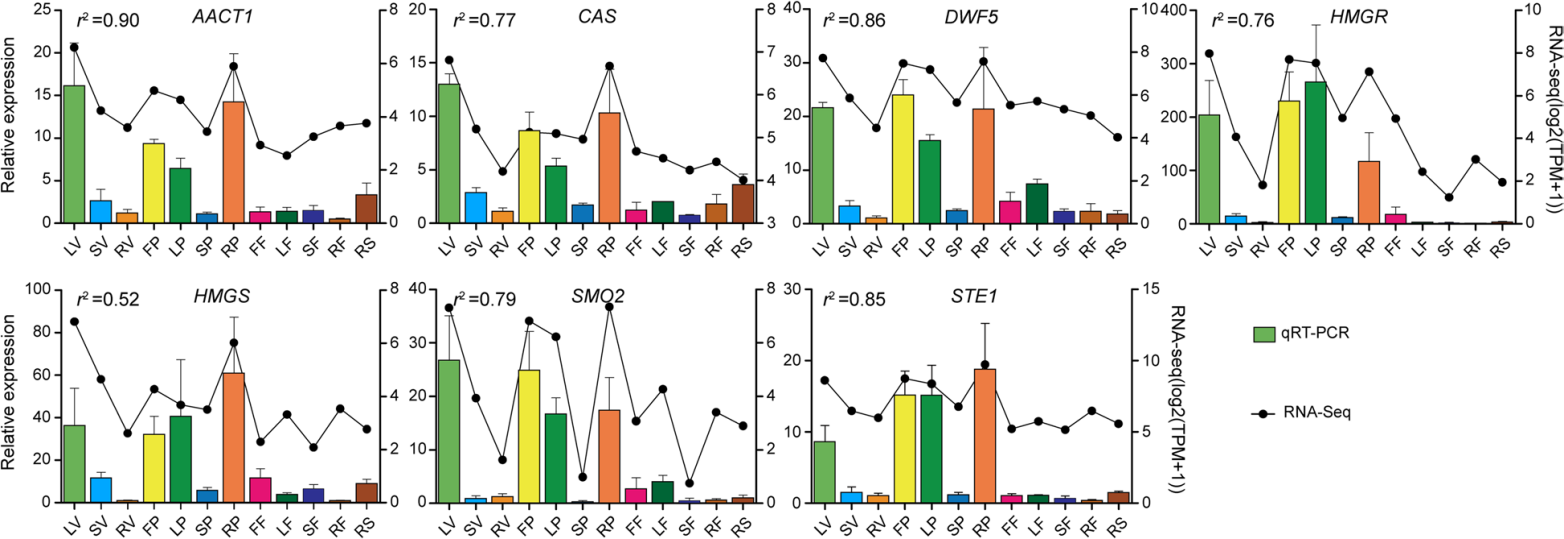
qRT-PCR validation of seven PBGs identified by RNA-Seq. The correlation is quantified using the Pearson correlation coefficient (*r*^*2*^)

To confirm the gene transcript levels in different tissues, seven DEGs from the predicted PBG candidates were selected for preforming qRT-PCR validation (Fig. 8). The primers used for qRT-PCR are listed in Additional file 1 Table S3. As expected, the expression levels of PBGs were consistent with the expression profiles derived from RNA-Seq results. The expression fold change values were also similar to the transcriptomic analysis results. Furthermore, the correlation calculation showed the positive correlation of RNA-Seq data with the qRT-PCR results (*r*^*2*^ > 0.50). Notably, the PBGs identified from the leaves at the vegetative and pollination stages, and from the flowers and the rhizomes during pollination stage were highly expressed, further corroborating the results of the transcriptome analysis. Thus, similar expression profiles and the high correlation value indicate the reliability and accuracy of the obtained RNA-Seq data.

## Discussion

### Polyphyllin biosynthesis in tissues is closely associated with plant development

Plant biosynthesis of specialized compounds, which often exclusively occurs in response to environmental stresses, is limited to dedicated anatomical structures [25]. Steroidal saponins have a broad spectrum of biological properties, such as antifungal, insecticidal, anti-herbivore, phytotoxic, and allelopathic effects [28]. For example, polyphyllins, with a multitude of antimicrobial and antiherbivore activities, are involved in defense in response to environmental cues [19]. In this study, polyphyllins were found to be unevenly distributed in the reproductive and vegetative tissues of *P. polyphylla*, implying that plant part substitution is a potential way to conserve the endangered medicinal plants [29]. Furthermore, the polyphyllin concentrations in different tissues significantly varied with plant development. Generally, the synthesis and accumulation of specialized plant metabolites are strictly controlled spatiotemporally [30]. Hence, the decreasing trends of polyphyllin concentration in the aerial vegetative tissues (leaves and stems) indicate that polyphyllins probably act as key deterrents against herbivore damage and microbial infections during the vegetative and pollination stages. However, polyphyllin biosynthesis gradually declined during leaf and stem ontogeny, which was confirmed using subsequent RNA-Seq analysis. In contrast, polyphyllin excessively accumulated in subterraneous rhizomes during pollination stage and remained relatively constant till the end of the annual growth cycle. This suggests the adaptions of this herb to specific ecological niches. In particular, *P. polyphylla* must adapt to dynamic environment to ensure optimal performance and eventual survival in competition with other individuals [25]. In most cases, the natural bioactive compounds found in medicinal plants are often seasonally regulated [31]. The synthesis of bioactive compounds in other medicinal plants is usually dynamic during development and occurs in different tissues [32–34]. Furthermore, both in situ biosynthesis and transport phenomena play roles in the accumulation of bioactive compounds in plant tissues [35].

Next-generation sequencing has greatly advanced the research on the important metabolic pathways and related enzymes in non-model plants [36]. We initially identified and functionally characterized 137 PBGs encoding the dominant enzymes in the upstream pathway of polyphyllin biosynthesis using a comprehensive set of RNA-Seq data. More genes from MVA pathway were detected in polyphyllin-related modules and expression patterns than in the MEP pathway, highlighting the predominant role of the MVA pathway in polyphyllin biosynthesis. This is in accordance with the results of most sterols and triterpenes, which are considered to be synthesized via the MVA pathway [37]. In contrast, the MEP pathway plays a pivotal role in camptothecin biosynthesis in *Nothapodytes nimmoniana* [38]. In *Panax ginseng*, the MEP pathway has a similar role in ginsenoside biosynthesis in ginseng roots and leaves [39]. Notably, almost all the identified PBGs were expressed in all the key vegetative tissues, indicating that the entire plant continuously participated in polyphyllin biosynthesis during development. In short, our results provide insights into the relationships between plant development and polyphyllin biosynthesis.

### Polyphyllin is dependent on the combined interplay of PBGs, TFs and transporters

The onset of specialized metabolite production is predominantly transcriptionally regulated by the action of specific TFs [25]. Therefore, polyphyllin accumulation may be regulated by a series of biosynthesis- and transport-related structural genes. Prior to this study, there was no co-expression network analysis-based data available for either the PBG-related transcriptional regulators or polyphyllin-related transporters. Among the numerous TFs, a set of 74 TF-coding genes were found to be closely associated with PBGs that were screened for the first time using GCNA. GCNA has been previously applied in various biological contexts and provides vital insights into plant secondary metabolism [40]. Notably, different TFs regulated the same target PBG, suggesting that the TFs form a regulatory complex. Additionally, one TF may have more than one target PBG; for example, the genes of the MYB family are involved in the MVA pathway and also the subsequent steps of polyphyllin biosynthesis. A combinatorial role of some TFs seems a plausible strategy for the regulation of biosynthetic pathway [41]. Specific TFs are often capable of coordinating the transcription of multiple biosynthetic genes, rendering them particularly effective in metabolic pathway engineering [42]. The specific genes encoding TFs AP2/ERF, bZIP [43], bHLH [44], MYB [45], and NAC [46] have been identified and verified to regulate the biosynthesis of different terpenes. However, the identity of the TF arrays in most terpenoid pathway, especially those closely associated with jasmonic acid (JA)-induced genes, has remained unclear [41]. Currently, the regulation mechanisms underlying polyphyllin biosynthesis remain obscure and require extensive effort to explore them. Our findings suggest that key TFs affect polyphyllin yield by regulating its metabolism in *P. polyphylla*, providing a reference for future research on polyphyllin regulation.

In addition, the genes responsible for synthesizing metabolites may be highly expressed in the tissues where metabolites are mainly stored, while translocation of natural compounds among plant organs also often occurs [31]. Through GCNA, we screened 13 transporter-coding genes belonging to GST, SLC, TMED, and ABC transmembrane transporter families that highly correlated with the identified PBGs. Notably, we discovered that the predicted *ABCB1* was closely associated with polyphyllin. *ABCB1* was highly expressed in the rhizomes during pollination and fruiting stages and in the leaves at the pollination stage. Plant ABC proteins are commonly classified into 13 subfamilies based on protein size (full or half), orientation (forward or reverse), idiotypic transmembrane or linker domains (presence or absence), and overall sequence similarity [47]. Interestingly, plants harboring mutations in ABC transporters exhibit deformed phenotypes, many of which are related to developmental processes and environmental adaptations [48]. For instance, the CjABCB1 localized at the plasma membrane of *Coptis japonica* is preferentially expressed in the xylem tissue of the rhizomes and catalyzes berberine translocation from the root to the rhizome to protect against pathogens [49]. ABC transporters are also involved in root exudation processes: one ABC transporter can transport structurally different compounds [50]. A pleiotropic drug resistance (PDR)-type ABC transporter in *Arabidopsis* and *Spirodela* can recognize sclareol or other natural compounds with similar structures and export them [51]. Thus, we speculate that ABCB1 is a crucial polyphyllin transporter for *P. polyphylla*. Taken together, GCNA utilization can facilitate the identification of transcription regulators and transporters that play a combinatorial role in regulating polyphyllin biosynthesis and accumulation. Additionally, we found that de novo transcriptome assembly of species with huge genome through next generation sequencing has some difficulties. The genome size of *P*.*polyphylla* was estimated over 50 Gb and it probably contains enormous homologs according to plant species with giant genome. In absence of the genome, billions of short reads generated from next generation sequencing bring the difficulties in transcriptome assembly. The data redundancy and fragments of genes inevitably occur and stand out in case of giant genome, absence of genome, and increasing sequence depth, though transcriptome sequencing quality was strictly controlled and the data cluttering and filtration analyses were conducted. These factors can influence the related ratios like proportion of protein-coding gene and noncoding gene. We will consider the Single-molecule, Real-time (SMRT) sequencing to reduce the impacts of technology and method on transcriptome assembly in the further research.

## Conclusions

Through phytochemical investigation, we discovered that polyphyllin accumulation in the tissues of *P. polyphylla* varied with plant development, and demonstrated polyphyllin accumulation in both the reproductive and vegetative tissues. The upstream pathway of polyphyllin biosynthesis was described, in which 137 putative PBGs were screened via RNA-Seq analysis. We proposed a model that summarized the spatiotemporal expressions of the identified PBGs. Moreover, the candidate TF and transporter genes closely associated with polyphyllin were mined. Our findings contribute to future research aimed at understanding the molecular mechanisms underlying polyphyllin biosynthesis, regulation and accumulation in *P. polyphylla*. Our study serves as a reference for exploring the medicinally and economically important ingredients in other medicinal plants.

## Methods

### Plant materials

The *P. polyphylla* var. *yunnanensis* plants in this study were obtained through seed germination and seedling cultivation for years, and the seeds were kindly provided by Yunnan Yuxin Agriculture and Forestry Biological Technology Co., Ltd. *P. polyphylla* var. *yunnanensis* plants were grown under the same planting management including light and water supplies, temperature and soil conditions in the green house of Xishuangbanna Tropical Botanical Garden, Chinese Academy of Sciences (Kunming). The fresh tissues including leaves, stems, rhizomes, flowers and fruits of the healthy seven-year-old plants were respectively harvested during the four developmental stages in a year. Each tissue sample was composed of the tissues from three different plants with the similar size, and each type of tissue was assigned with three biological replicates. The leaves, stems, and rhizomes were washed with tap water and ultrapure water respectively.

### Polyphyllin concentration determination

After cleaning with ultrapure water, the tissues prepared for phytochemical investigation were separately dried to achieve constant weight in the oven at 40℃ and they then were grinded into powder. The extraction and quantification of polyphyllins were performed as previously reported [24]. In detail, 0.5 g of the powder sample was exhaustively mixed with 70% EtOH (25 mL). The mixture was heated under reflux 30 min and added 70% EtOH for the weight loss after cool down. The supernatant was then filtered to get sample solution after centrifugation. Quantitative analysis of polyphyllins (Polyphyllin I, II, VI, and VII) were performed on an Agilent HPLC 1260 series system (Agilent, USA) and the retention time of the four types of polyphyllins were compared to that of the relative standard substances. The chromatographic separation was performed at temperature 30℃ using acetonitrile (A) and water (B) with gradient elution procedure (0–40 min, linear gradient 30–60% A, linear gradient 70–40% B; 40–50 min, linear gradient 60–30% A, linear gradient 40–70% B) as the mobile phase at a flow rate of 1.0 mL/min [52].

### RNA extraction, library construction and transcriptome sequencing

The tissues prepared for RNA-Seq were collected separately, and immediately frozen in liquid nitrogen, and finally stored at -80℃. Total RNA respectively extracted from different tissue samples, was treated with RNAse-Free DNAse and purified using RNasy (QIAGEN, Hilden, Germany). The quality and quantity of RNA was determined by agarose gel electrophoresis, NanoDrop 2000 spectrophotometer (Thermo Fisher Scientific, USA), and Aglient Bioanalyzer 2100 system (Agilent Technologies, USA). A total of 20 μg RNA from each sample was used for cDNA library preparation. The RNA molecules containing poly (A) from each total RNA sample were purified using oligo (dT) magnetic beads. The purified mRNA was first fragmented into small pieces with an average length of 155 bp (120–210 bp) at 94 °C for exactly 5 min. Taking these short mRNA fragments as templates, first-strand cDNAs were synthesized using random hexamer primers and reverse transcriptase. The second-strand cDNA was synthesized using Buffer, dNTPs, RNase H, and DNA polymerase. The cDNAs were then subjected to end-repair. The “A” base was inserted at the 3′ end to repair cDNAs, and adaptors were connected to these cDNA fragments to select different fragments. PCR amplification was performed to enrich cDNA library. Agilent 2100 Bioanaylzer and Qubit Fluorometer (Thermo Fisher Scientific Inc., Waltham, MA, USA) were used to assess the quality of cDNA library. An Illumina HiSeq™ 2500 platform with 125 bp paired-end reads was applied for transcriptome sequencing. Library construction and sequencing were performed by Vazyme Biotech Co., Ltd (Nanjing, China).

### De novo transcriptome assembling and functional annotation

The quality of the paired-end raw reads was checked using FastQC. The adapters, poly-A tails, primer sequences, low quality sequences with Phred quality scores Q < 20, and ambiguous sequences were removed. The clean reads were registered in the NCBI database with accession no. PRJNA682903 and they then were preprocessed and trimmed using Trimmomatic [53]. Combing with RNA-Seq data in our previous study (NCBI, accession no. PRJNA630028) [24], the high quality clean reads were assembled using Trinity v2.6.6 with min_kmer_cov set to 7 [54]. The accuracy and completeness of assembled transcriptome was evaluated using BUSCO v3.0.2 [55]. The unigene set was obtained by clustering the longest isoforms per each gene using CD-HIT and Corset [56, 57]. All the unigene sequences were subjected to similarity searching against Nr and KOG using BLAST with an E-value cutoff of 1e-5. Meanwhile, unigenes were functionally annotated by GO, Pfam, and Swiss-Prot using Trinotate pipeline [58]. Additionally, unigenes were mapped to KEGG pathways using KEGG Automatic Annotation Server (KAAS) [59]. The PBGs were extracted and counted. As the large genome size of *P. polyphyll*a and little known about genomes of the order Liliales [26, 27], the unannotated unigenes then subjected to noncoding RNA prediction by both CNCI and CPC2 [60, 61].

### Differential expression analysis

Gene expression levels were calculated and normalized as TPM approach using by RSEM [62]. Differential expressed genes (DEGs) were determined using DESeq2 package [63]. Adjusted *p* value < 0.01, $|{\mathrm{log}}_{2}(\mathrm{fold change})|\ge 2$ was set as the threshold for significantly differential expression. The valid DEG set (23,759) was composed of DEGs of 27 paired-comparison groups. GO and KEGG enrichment of DEGs were performed using GOseq R package and clusterProfiler R package [64, 65]. The *p* values were adjusted using Benjamini-Hochberg’s method [66]. Adjusted *p* value < 0.05 was set as a threshold to determine significant enrichment of these DEGs.

### Spatiotemporal gene expression patterns

Expression pattern of a specific tissue across different developmental stages was dissected. The DEG data sets of leaves (LV vs. LP, LV vs. LF, LP vs. LF), stems (SV vs. SP, SV vs. SF, SP vs. SF), and rhizomes (RV vs. RP, RV vs. RF, RV vs. RS, RP vs. RF, RP vs. RS, RF vs. RS) were extracted and built, respectively. Clustering analysis of expression levels of the three tissues was conducted by the fuzzy clustering algorithm using mfuzz R package [67]. All the clusters of different tissues were then subjected to KEGG enrichment analysis.

### Identification of the related genes co-expressed with PBGs

GCNA was carried out using GCEN v0.5.0 [68]. The correlation relationships of polyphyllin content and expression levels of DEGs were analyzed using WGCNA R package [69]. The transcriptional factor genes co-expressed with PBGs were counted. The networks of PBGs and TF genes were visualized using Cytcoscape v3.7.2 [70]. Additionally, transporter genes co-expressed with PBGs were identified. Correlation analysis between expression profiles of transporter genes and polyphyllin concentration was perform with R package corrplot [71].

###  qRT-PCR analysis

Total RNA was extracted from the tissues as above. The first-strand cDNA was synthesized from 1 μg of total RNA by using the RvertAid™ First Strand cDNA Synthesis Kit (Thermo Scientific, USA). Seven PBGs were selected for qRT-PCR to verify the RNA-Seq data. The primers of these genes were designed using Primer Premier v5.0. qRT-PCRs were performed using TB Green Premix Ex Taq II (Tli RNase H Plus), and qRT-PCR analysis was run on a CFX96 TouchTM Real-Time PCR Detection System (BioRad, America) with CFX96 TouchTM Optics Module. All of the PCR reactions were conducted in triplicated, and the average expression values are means of three biological replicates of each sample. The target genes were normalized to an internal standard *Actin* shown as ${2}^{-\Delta \Delta \mathrm{Ct}}$ [72]. The correlation tests between the qRT-PCR and RNA-data were performed using Pearson method.

## Supplementary Information

**Additional file 1:** This Excel file contains the additional tables (Table S1-S3) associated with the manuscript. Table numbers and titles are listed as follows: **Table S1.** Summary of transcriptome sequencing data generated and analyzed in this study. **Table S2.** Functional annotation of unigenes. **Table S3. **Primer sets of selected PBGs used for qRT-PCR analysis.

**Additional file 2:** This PDF contains all the additional figures (Figure S1-S6) associated with the manuscript. Figure numbers and titles are listed as follows: **Figure S1.** BUSCO assessment results. **Figure S2.** KEGG enrichment analysis of DEGs from 27 paired groups. **Figure S3.** Ten cluster of DEGs in leaf and the relative KEGG enrichments. **Figure S4.** Ten cluster of DEGs in stem and the relative KEGG enrichments. **Figure S5.** Ten cluster of DEGs in rhizome and the relative KEGG enrichments. **Figure S6.** Module-tissue association from WGCNA analysis and KEGG enrichments of modules with affinity for polyphyllin.

**Additional file 3.** This compressed file contains the assembled transcriptome sequence in fasta format.

## Data Availability

The datasets supporting the conclusions of this article are available in the NCBI with accession number PRJNA682903 and PRJNA630028. The dataset supporting the conclusions of this article is included within its additional files (Additional file 3).

## Abbreviations

ABC: ATP-binding cassette
CAS: Cycloartenol synthase
DEGs: Differentially expressed genes
GCNA: Gene co-expression network analysis
GST: Glutathione S-transferase
HPLC: High-performance liquid chromatography
KAAS: KEGG Automatic Annotation Server
MVA: Cytosolic mevalonate
MEP: 2-C-methyl-D-erythritol 4-phospate
P450: Cytochrome P450 monooxygenase
PBGs: Polyphyllin biosynthetic genes
PDR: Pleiotropic drug resistance
qRT-PCR: Quantitative reverse transcription PCR
SLC: Solute carrier
TCM: Traditional Chinese medicine
TF: Transcription factor
TMED: Transmembrane Emp24 domain-containing protein

## References

[1] Li H (2008). The genus *Paris L*.

[2] Chase MW, Christenhusz MJM, Fay MF, Byng JW, Judd WS, Soltis DE, Mabberley DJ, Sennikov AN, Soltis PS, Stevens PF (2016). An update of the angiosperm phylogeny group classification for the orders and families of flowering plants: APG IV. Bot J Linn Soc.

[3] Liu Z, Li N, Gao WY, Man SL, Yin SS, Liu CX (2012). Comparative study on hemostatic, cytotoxic and hemolytic activities of different species of *Paris* L. J Ethnopharmacol.

[4] Cunningham AB, Brinckmann JA, Bi YF, Pei SJ, Schippmann U, Luo P (2018). *Paris* in the spring: A review of the trade, conservation and opportunities in the shift from wild harvest to cultivation of *Paris polyphylla* (Trilliaceae). J Ethnopharmacol.

[5] Wu X, Wang L, Wang GC, Wang H, Dai Y, Yang XX, et al. Triterpenoid saponins from rhizomes of *Paris polyphylla* var. *yunnanensis*. Carbohyd Res. 2013;368:1–7. 10.1016/j.carres.2012.11.027 23305676

[6] Wei JC, Gao WY, Yan XD, Wang Y, Jing SS, Xiao PG (2014). Chemical constituents of plants from the genus *Paris*. Chem Biodivers.

[7] Committee of National Pharmacopoeia (2015). Pharmacopoeia of the People's Republic of China (I), 2015ed.

[8] Gao XY, Zhang X, Meng HH, Li J, Zhang D, Liu CN. Comparative chloroplast genomes of *Paris* Sect. *Marmorata*: insights into repeat regions and evolutionary implications. BMC Genomics. 2018;19(Suppl 10):133–44. 10.1186/s12864-018-5281-x PMC6311911 30598104

[9] Qin XJ, Chen CX, Ni W, Yan H, Liu HY. C-22-steroidal lactone glycosides from stems and leaves of *Paris polyphylla* var. *yunnanensis*. Fitoterapia. 2013;84:248–51. 10.1016/j.fitote.2012.12.007 23262268

[10] Qiang Q, Gao YF, Yu BZ, Wang ML, Ni W, Li SH, et al. Elevated CO2 enhances growth and differentially affects saponin content in *Paris polyphylla* var. *yunnanensis*. Ind Crop Prod. 2020;147:112124.

[11] Raomai S, Kumaria S, Tandon P (2014). Plant regeneration through direct somatic embryogenesis from immature zygotic embryos of the medicinal plant, *Paris polyphylla* Sm. Plant Cell Tiss Org.

[12] Zhang T, Liu H, Liu XT, Xu DR, Chen XQ, Wang Q. Qualitative and quantitative analysis of steroidal saponins in crude extracts from *Paris polyphylla *var. *yunnanensis* and *P. polyphylla* var. *chinensis *by high performance liquid chromatography coupled with mass spectrometry. J Pharmaceut Biomed. 2010;51(1):114–24. 10.1016/j.jpba.2009.08.020 19767166

[13] Madhav K, Phoboo S, Jha PK (2010). Ecological study of *Paris polyphylla* Sm. Int J Ecol.

[14] Negi JS, Bisht VK, Bhandari AK, Bhatt VP, Singh P, Singh N (2014). *Paris polyphylla*: Chemical and biological prospectives. Anti-Cancer Agent Me.

[15] Qin XJ, Yu MY, Ni W, Yan H, Chen CX, Cheng YC, et al. Steroidal saponins from stems and leaves of *Paris polyphylla* var. *yunnanensis*. Phytochemistry. 2016;121:20–9. 10.1016/j.phytochem.2015.10.008 26546502

[16] Liu T, Li X, Xie S, Wang L, Yang S. RNA-seq analysis of *Paris polyphylla* var. *yunnanensis *roots identified candidate genes for saponin synthesis. Plant Divers. 2016;38(3):163–70. 10.1016/j.pld.2016.05.002 PMC6112097 30159461

[17] Li B, Peng L, Sun XC, Huang WJ, Wang N, He YH, Shi XB, Liu YR, Zhang P, Yang XJ (2020). Organ-specific transcriptome sequencing and mining of genes involved in polyphyllin biosynthesis in *Paris polyphylla*. Ind Crop Prod.

[18] Yang ZY, Yang LF, Liu CK, Qin XJ, Liu HY, Chen JH, et al. Transcriptome analyses of *Paris polyphylla* var. *chinensis*, *Ypsilandra thibetica*, and *Polygonatum kingianum *characterize their steroidal saponin biosynthesis pathway. Fitoterapia. 2019;135:52–63. 10.1016/j.fitote.2019.04.008 30999023

[19] Christ B, Xu C, Xu M, Li FS, Wada N, Mitchell AJ, Han XL, Wen ML, Fujita M, Weng JK (2019). Repeated evolution of cytochrome P450-mediated spiroketal steroid biosynthesis in plants. Nat Commun.

[20] Yin Y, Gao LH, Zhang XN, Gao W (2018). A cytochrome P450 monooxygenase responsible for the C-22 hydroxylation step in the *Paris polyphylla* steroidal saponin biosynthesis pathway. Phytochemistry.

[21] Guo SY, Yin Y, Lei T, Shi YH, Gao W, Zhang XN, Li J (2020). A cycloartenol synthase from the steroidal saponin biosynthesis pathway of *Paris polyphylla*. J Asian Nat Prod Res.

[22] Wu Z, Zhang J, Xu FR, Wang YZ, Zhang JY. Rapid and simple determination of polyphyllin I, II, VI, and VII in different harvest times of cultivated *Paris polyphylla* Smith var. *yunnanensis *(Franch.) Hand.-Mazz by UPLC-MS/MS and FT-IR. J Nat Med-Tokyo. 2017;71(1):139–47. 10.1007/s11418-016-1043-8 27665608

[23] Wang YZ, Li P. Effect of cultivation years on saponins in* Paris Polyphylla* var. *yunnanensis *using ultra-high liquid chromatography-tandem mass spectrometry and Fourier transform infrared spectroscopy. Plant Growth Regul. 2018;84(2):373–81.

[24] Gao XY, Zhang X, Chen W, Li J, Yang WJ, Zhang XW, et al. Transcriptome analysis of *Paris polyphylla *var *yunnanensis *illuminates the biosynthesis and accumulation of steroidal saponins in rhizomes and leaves. Phytochemistry. 2020;178:112460. 10.1016/j.phytochem.2020.112460 32692662

[25] Colinas M, Goossens A (2018). Combinatorial Transcriptional Control of Plant Specialized Metabolism. Trends Plant Sci.

[26] Pellicer J, Kelly LJ, Leitch IJ, Zomlefer WB, Fay MF (2014). A universe of dwarfs and giants: genome size and chromosome evolution in the monocot family Melanthiaceae. New Phytol.

[27] Li J, Lv MQ, Du L, Yunga A, Hao SJ, Zhang YL, et al. An enormous *Paris polyphylla* genome sheds light on genome size evolution and polyphyllin biogenesis. BioRxiv. 2020. preprint.

[28] Waller GR, Yamasaki K (2013). Saponins used in traditional and modern medicine.

[29] Zschocke S, Rabe T, Taylor JLS, Jager AK, van Staden J (2000). Plant part substitution-a way to conserve endangered medicinal plants?. J Ethnopharmacol.

[30] Patra B, Schluttenhofer C, Wu Y, Pattanaik S, Yuan L (2013). Transcriptional regulation of secondary metabolite biosynthesis in plants. Biochim Biophys Acta.

[31] Yazaki K (2006). ABC transporters involved in the transport of plant secondary metabolites. Febs Lett.

[32] Schmitt B, Schulz H, Storsberg J, Keusgen M. Chemical characterization of* Allium ursinum* L. depending on harvesting time. J Agr Food Chem. 2005;53(18):7288–94. 10.1021/jf0504768 16131144

[33] Lubbe A, Gude H, Verpoorte R, Choi YH (2013). Seasonal accumulation of major alkaloids in organs of pharmaceutical crop *Narcissus Carlton*. Phytochemistry.

[34] Ji M, Li Q, Ji H, Lou HX (2014). Investigation of the distribution and season regularity of resveratrol in *Vitis amurensis* via HPLC-DAD-MS/MS. Food Chem.

[35] del Bano MJ, Lorente J, Castillo J, Benavente-Garcia O, del Rio JA, Ortuno A, Quirin KW, Gerard D (2003). Phenolic diterpenes, flavones, and rosmarinic acid distribution during the development of leaves, flowers, stems, and roots of *Rosmarinus officinalis*. Antioxidant activity J Agr Food Chem.

[36] Xiao M, Zhang Y, Chen X, Lee EJ, Barber CJS, Chakrabarty R, Desgagne-Penix I, Haslam TM, Kim YB, Liu EW (2013). Transcriptome analysis based on next-generation sequencing of non-model plants producing specialized metabolites of biotechnological interest. J Biotechnol.

[37] Thimmappa R, Geisler K, Louveau T, O'Maille P, Osbourn A (2014). Triterpene Biosynthesis in Plants. Annu Rev Plant Biol.

[38] Rather GA, Sharma A, Jeelani SM, Misra P, Kaul V, Lattoo SK (2019). Metabolic and transcriptional analyses in response to potent inhibitors establish MEP pathway as major route for camptothecin biosynthesis in *Nothapodytes nimmoniana* (Graham) Mabb. BMC Plant Biol.

[39] Xue L, He ZL, Bi XC, Xu W, Wei T, Wu SX, Hu SN (2019). Transcriptomic profiling reveals MEP pathway contributing to ginsenoside biosynthesis in *Panax ginseng*. BMC Genomics.

[40] Higashi Y, Saito K (2013). Network analysis for gene discovery in plant-specialized metabolism. Plant Cell Environ.

[41] Van Moerkercke A, Steensma P, Schweizer F, Pollier J, Gariboldi I, Payne R, Vanden Bossche R, Miettinen K, Espoz J, Purnama PC (2015). The bHLH transcription factor BIS1 controls the iridoid branch of the monoterpenoid indole alkaloid pathway in *Catharanthus roseus*. Proc Natl Acad Sci U S A.

[42] Suttipanta N, Pattanaik S, Kulshrestha M, Patra B, Singh SK, Yuan L (2011). The transcription factor CrWRKY1 positively regulates the terpenoid indole alkaloid biosynthesis in *Catharanthus roseus*. Plant Physiol.

[43] Zhang Y, Ji AJ, Xu ZC, Luo HM, Song JY (2019). The AP2/ERF transcription factor SmERF128 positively regulates diterpenoid biosynthesis in *Salvia miltiorrhiza*. Plant Mol Biol.

[44] Van Moerkercke A, Steensma P, Gariboldi I, Espoz J, Purnama PC, Schweizer F, Miettinen K, Vanden Bossche R, De Clercq R, Memelink J (2016). The basic helix-loop-helix transcription factor BIS2 is essential for monoterpenoid indole alkaloid production in the medicinal plant *Catharanthus roseus*. Plant J.

[45] Shen Q, Zhang LD, Liao ZH, Wang SY, Yan TX, Shi P, Liu M, Fu XQ, Pan QF, Wang YL (2018). The genome of *Artemisia annua* provides insight into the evolution of Asteraceae family and artemisinin biosynthesis. Mol Plant.

[46] Nieuwenhuizen NJ, Chen X, Wang MY, Matich AJ, Perez RL, Allan AC, Green SA, Atkinson RG (2015). Natural variation in monoterpene synthesis in kiwifruit: transcriptional regulation of terpene synthases by NAC and ETHYLENE-INSENSITIVE3-like transcription factors. Plant Physiol.

[47] Sanchez-Fernandez R, Davies TGE, Coleman JOD, Rea PA (2001). The *Arabidopsis thaliana* ABC protein superfamily, a complete inventory. J Biol Chem.

[48] Hwang JU, Song WY, Hong D, Ko D, Yamaoka Y, Jang S, Yim S, Lee E, Khare D, Kim K (2016). Plant ABC transporters enable many unique aspects of a terrestrial plant's lifestyle. Mol Plant.

[49] Shitan N, Bazin I, Dan K, Obata K, Kigawa K, Ueda K, Sato F, Forestier C, Yazaki K (2003). Involvement of CjMDR1, a plant multidrug-resistance-type ATP-binding cassette protein, in alkaloid transport in *Coptis japonica*. Proc Natl Acad Sci U S A.

[50] Badri DV, Vivanco JM (2009). Regulation and function of root exudates. Plant Cell Environ.

[51] van den Brule S, Muller A, Fleming AJ, Smart CC (2002). The ABC transporter SpTUR2 confers resistance to the antifungal diterpene sclareol. Plant J.

[52] Man S, Gao W, Zhang Y, Wang J, Zhao W, Huang L, Liu C (2010). Qualitative and quantitative determination of major saponins in *Paris* and *Trillium* by HPLC-ELSD and HPLC-MS/MS. J Chromatogr B.

[53] Bolger AM, Lohse M, Usadel B (2014). Trimmomatic: a flexible trimmer for Illumina sequence data. Bioinformatics.

[54] Grabherr MG, Haas BJ, Yassour M, Levin JZ, Thompson DA, Amit I, Adiconis X, Fan L, Raychowdhury R, Zeng QD (2011). Full-length transcriptome assembly from RNA-Seq data without a reference genome. Nat Biotechnol.

[55] Simao FA, Waterhouse RM, Ioannidis P, Kriventseva EV, Zdobnov EM (2015). BUSCO: assessing genome assembly and annotation completeness with single-copy orthologs. Bioinformatics.

[56] Fu LM, Niu BF, Zhu ZW, Wu ST, Li WZ (2012). CD-HIT: accelerated for clustering the next-generation sequencing data. Bioinformatics.

[57] Davidson NM, Oshlack A (2014). Corset: enabling differential gene expression analysis for de novo assembled transcriptomes. Genome Biol.

[58] Grabherr MG, Haas BJ, Yassour M, Levin JZ, Thompson DA, Amit I, Adiconis X, Fan L, Raychowdhury R, Zeng Q (2011). Full-length transcriptome assembly from RNA-Seq data without a reference genome. Nat Biotechnol..

[59] Moriya Y, Itoh M, Okuda S, Yoshizawa AC, Kanehisa M. KAAS: an automatic genome annotation and pathway reconstruction server. Nucleic Acids Res. 2007;35(suppl_2):W182–5. 10.1093/nar/gkm321 PMC1933193 17526522

[60] Sun L, Luo HT, Bu DC, Zhao GG, Yu KT, Zhang CH, Liu YN, Chen RS, Zhao Y (2013). Utilizing sequence intrinsic composition to classify protein-coding and long non-coding transcripts. Nucleic Acids Res.

[61] Kang YJ, Yang DC, Kong L, Hou M, Meng YQ, Wei LP, Gao G (2017). CPC2: a fast and accurate coding potential calculator based on sequence intrinsic features. Nucleic Acids Res.

[62] Li B, Dewey CN (2011). RSEM: accurate transcript quantification from RNA-Seq data with or without a reference genome. BMC Bioinformatics.

[63] Love MI, Huber W, Anders S (2014). Moderated estimation of fold change and dispersion for RNA-seq data with DESeq2. Genome Biol.

[64] Young MD, Wakefield MJ, Smyth GK, Oshlack A (2010). Gene ontology analysis for RNA-seq: accounting for selection bias. Genome Bio.

[65] Yu GC, Wang LG, Han YY, He QY (2012). clusterProfiler: an R Package for Comparing Biological Themes Among Gene Clusters. OMICS.

[66] Benjamini Y, Hochberg Y (1995). Controlling the false discovery rate-a practical and powerful approach to multiple testing. J R Stat Soc B.

[67] Kumar LMEF (2007). Mfuzz: a software package for soft clustering of microarray data. Bioinformation.

[68] Chen W, Li J, Huang SL, Li XD, Zhang X, Hu X, Xiang SL, Liu CN (2022). GCEN: An Easy-to-Use Toolkit for Gene Co-Expression Network Analysis and lncRNAs Annotation. Curr Issues Mol Biol.

[69] Langfelder P, Horvath S (2008). WGCNA: an R package for weighted correlation network analysis. BMC Bioinformatics.

[70] Shannon P, Markiel A, Ozier O, Baliga NS, Wang JT, Ramage D, Amin N, Schwikowski B, Ideker T (2003). Cytoscape: A software environment for integrated models of biomolecular interaction networks. Genome Res.

[71] Wei T, Simko V, Levy M, Xie Y, Jin Y, Zemla J (2017). Package ‘corrplot’. Stat.

[72] Schmittgen TD (2008). Livak KJ Analyzing real-time PCR data by the comparative C-T method. Nat Protoc.

