# Supplementary material for: RNA-Seq analysis reveals the important co-expressed genes associated with polyphyllin biosynthesis during the developmental stages of Paris polyphylla
Source: BMC Genomics. 2022 Aug 5;23(Suppl 1):559. doi: 10.1186/s12864-022-08792-2 (PMC9354290; doi:10.1186/s12864-022-08792-2)
Supplement: Supplementary file 2 — Additional file 2: This PDF contains all the additional figures (Figure S1-S6) associated with the manuscript. Figure numbers and titles are listed as follows: Figure S1. BUSCO assessment results. Figure S2. KEGG enrichment analysis of DEGs from 27 paired groups. Figure S3. Ten cluster of DEGs in leaf and the relative KEGG enrichments. Figure S4. Ten cluster of DEGs in stem and the relative KEGG enrichments. Figure S5. Ten cluster of DEGs in rhizome and the relative KEGG enrichments. Figure S6. Module-tissue association from WGCNA analysis and KEGG enrichments of modules with affinity for polyphyllin. [file 12864_2022_8792_MOESM2_ESM.pdf]

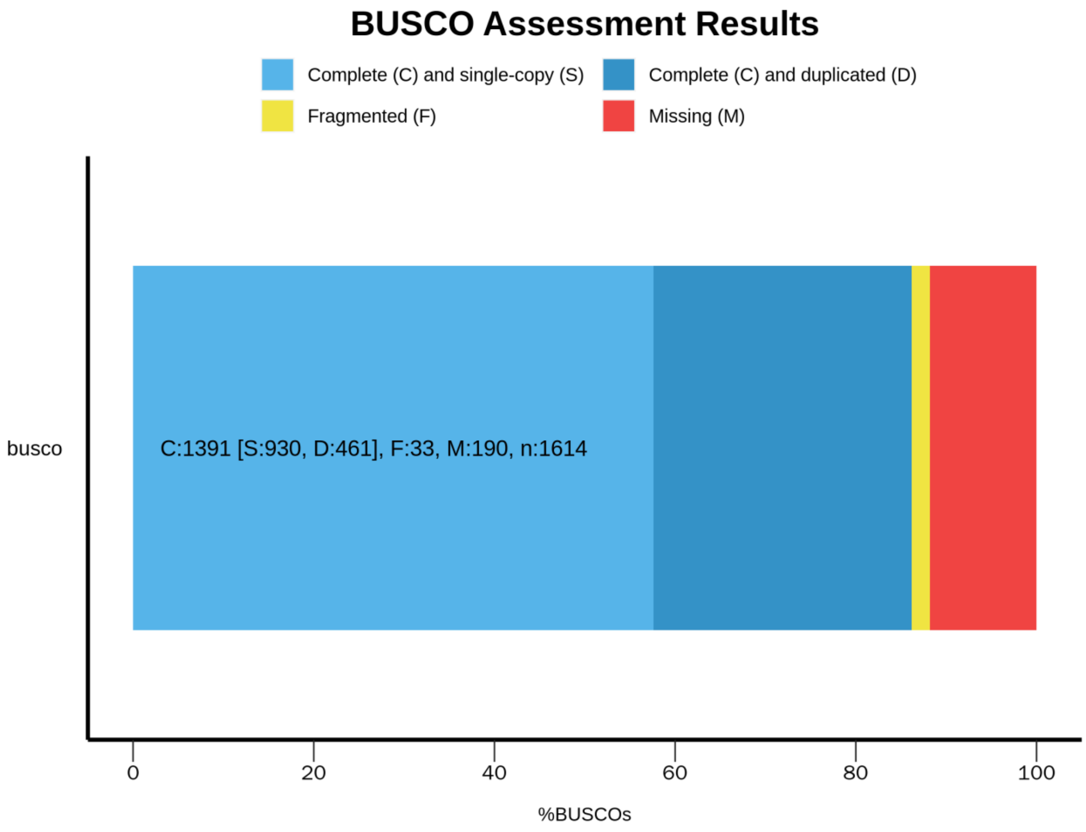

Figure S1 BUSCO assessment results.

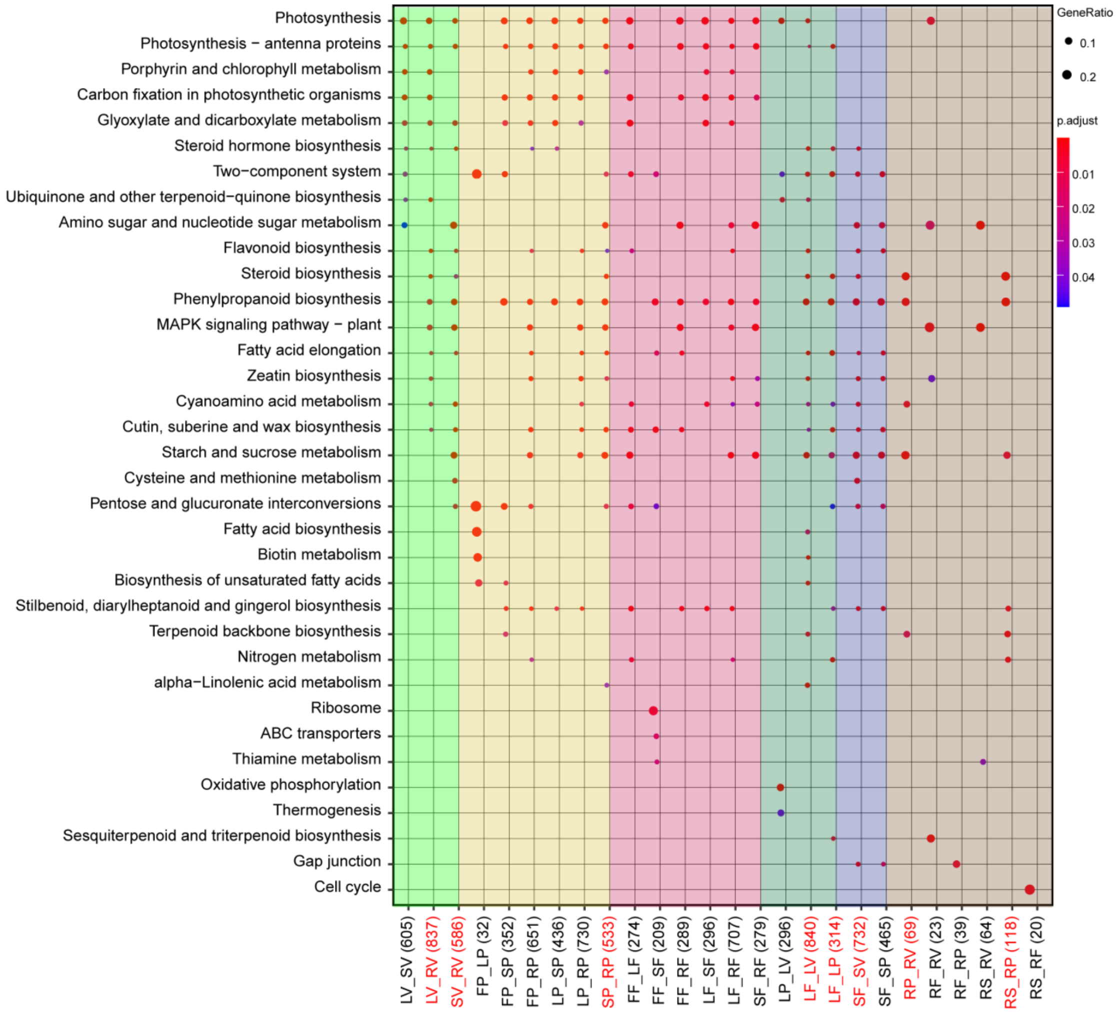

Figure S2 KEGG enrichment analysis of DEGs from 27 paired groups.

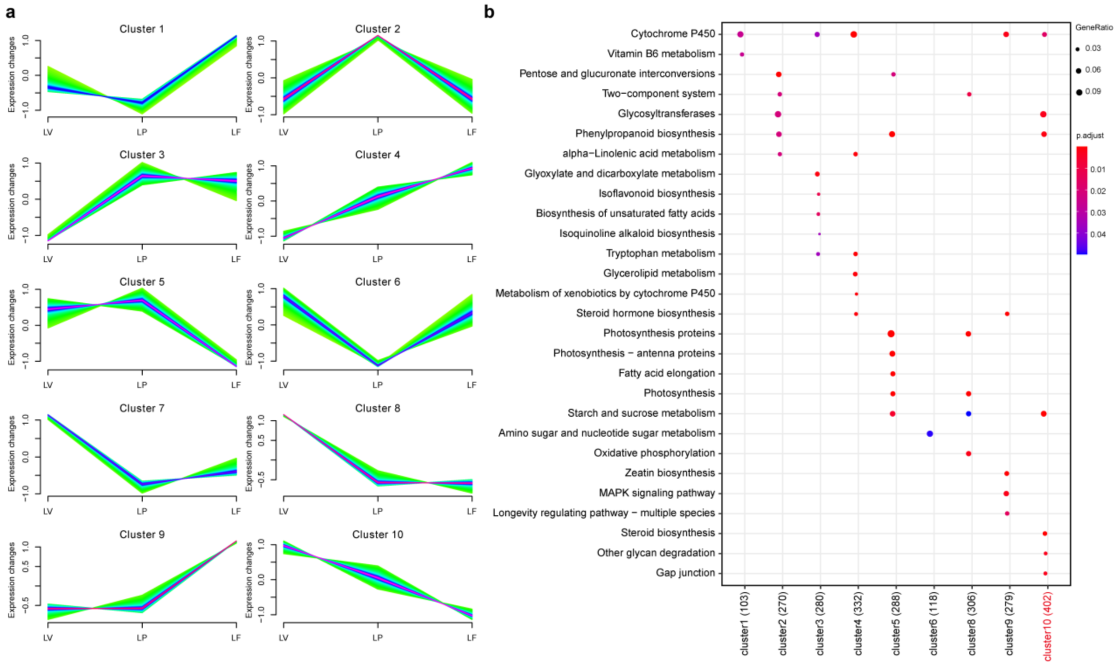

Figure S3 Ten cluster of DEGs in leaf and the relative KEGG enrichments.

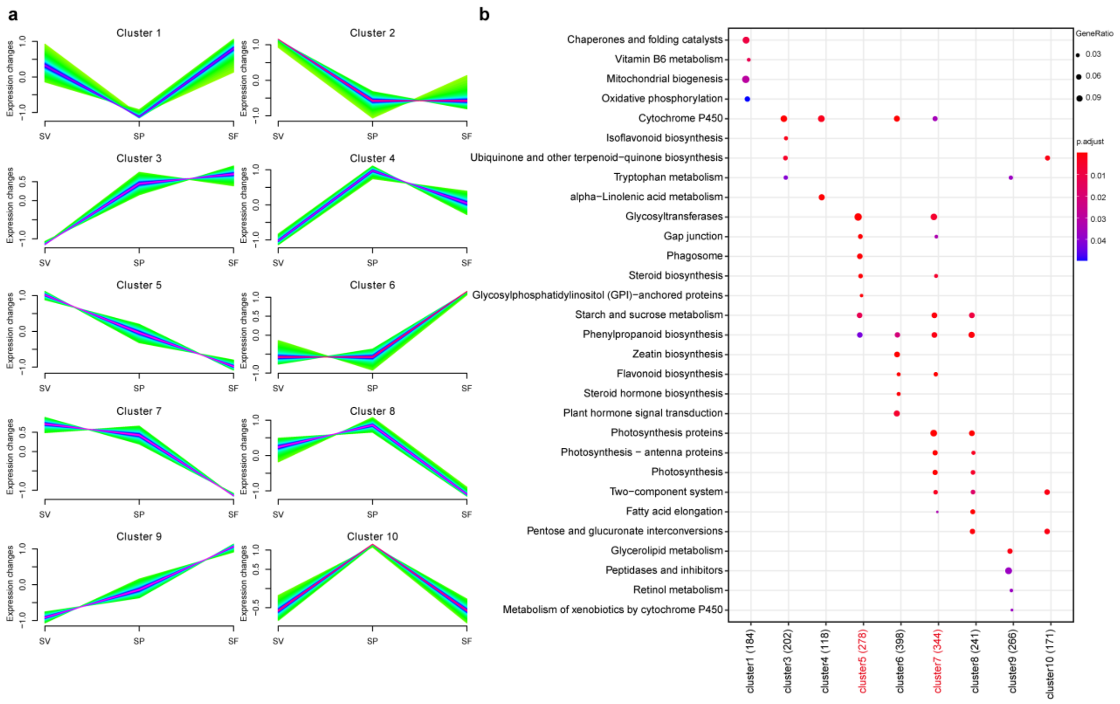

Figure S4 Ten cluster of DEGs in stem and the relative KEGG enrichments.

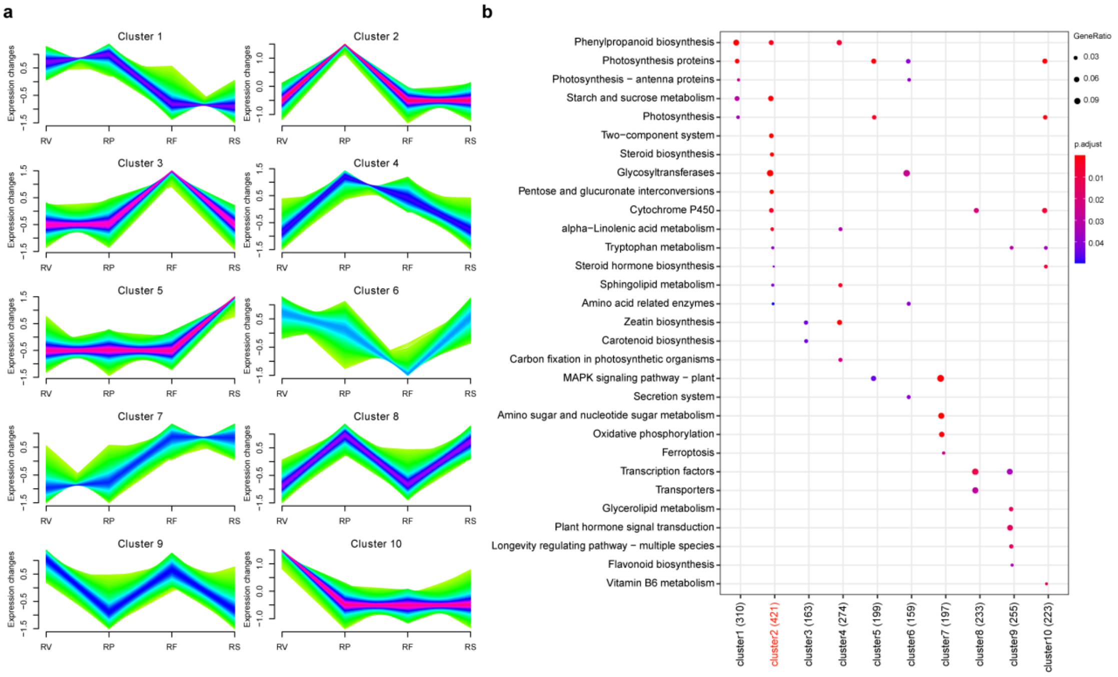

Figure S5 Ten cluster of DEGs in rhizome and the relative KEGG enrichments.

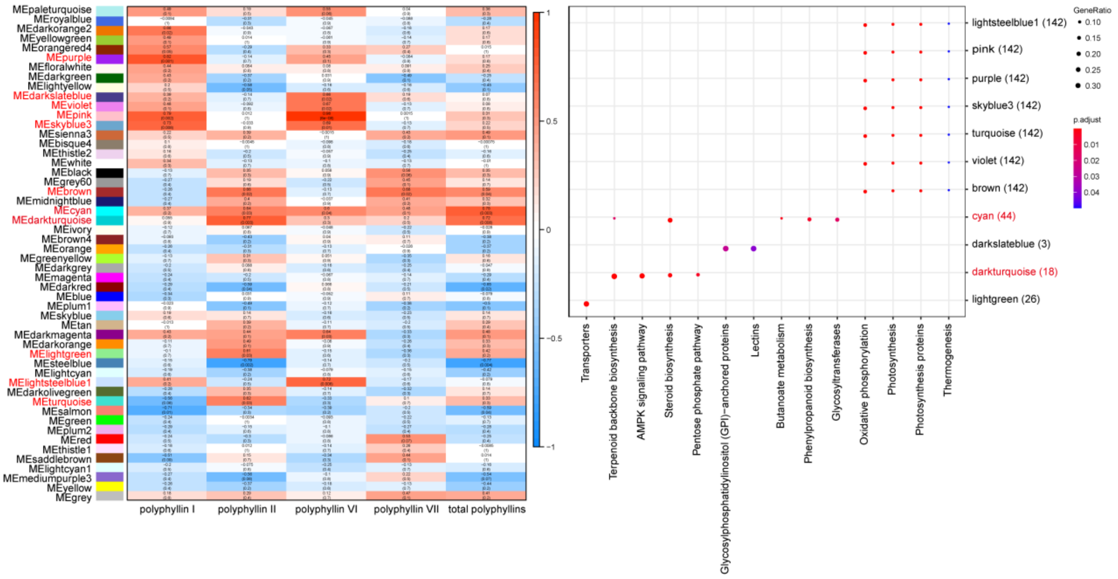

Figure S6 Module-tissue association from WGCNA analysis and KEGG enrichments of modules with affinity for polyphyllin.
